# Lifetime and Past-Year Defensive Gun Use

**DOI:** 10.1001/jamanetworkopen.2025.0807

**Published:** 2025-03-14

**Authors:** Michael D. Anestis, Kimberly Burke, Sultan Altikriti, Daniel Semenza

**Affiliations:** 1 New Jersey Gun Violence Research Center, Piscataway; 2 School of Public Health, Rutgers, State University of New Jersey, Piscataway; 3 Department of Sociology, Anthropology, and Criminal Justice, Rutgers University, Camden, New Jersey

## Abstract

**Importance:**

Defensive gun use (DGU) is cited as a rationale for permissive firearm-carrying policies; however, no consensus exists on how frequently DGU occurs.

**Objective:**

To examine the frequency of DGU relative to gun violence exposure (GVE) in a sample of firearm owners drawn from a nationally representative sample of US adults.

**Design, Setting, and Participants:**

This survey study used data from a cross-sectional, self-reported survey administered via KnowledgePanel, a probability-based panel, between May 15 and May 28, 2024. Eligible participants were adults residing within the US reporting current firearm access who responded to DGU survey items. Data were analyzed from July to September 2024.

**Main Outcomes and Measures:**

Primary outcomes were 4 forms of DGU: telling a perceived threat about a firearm, showing a firearm to a perceived threat, firing in the vicinity of but not at a perceived threat, and firing at a perceived threat.

**Results:**

Among 12 822 adults invited to participate, 8647 (67.4%) read the informed consent, 8009 (92.6%) consented to participate, and 3000 (37.7%; 532 [51.1%] male; 982 [32.7%] aged ≥60 years) endorsed firearm access and responded to DGU items, including 295 Black, non-Hispanic participants (9.8%); 365 Hispanic participants (12.2%); and 2178 White, non-Hispanic participants (72.6%). DGU was rare, with 91.7% (95% CI, 90.6%-92.7%) of participants reporting no lifetime history of DGU. The most reported form of DGU was showing a firearm to a perceived threat (lifetime: 4.7%; 95% CI, 4.0%-5.5%). Less than 1% of the sample reported any form of past-year DGU. GVE was more pervasive for lifetime (eg, loss of a friend or loved one to firearm suicide: 34.4%; 95% CI, 32.7%-36.1%; hearing gunshots in neighborhood: 51.8%; 95% CI, 50.0%-53.6%) and past-year exposure (eg, loss of a friend or loved one to firearm suicide: 3.2%; 95% CI, 2.6%-3.9%; hearing gunshots in neighborhood: 32.7%; 95% CI, 31.0%-34.4%).

**Conclusions and Relevance:**

In this survey of adults with firearm access, DGU was rare relative to GVE. Perceived threats may not necessitate DGU, and given the association between DGU and GVE, the consequences of DGU may be substantial. Narratives centering DGU as a consideration in firearm policies may misstate the risk profile of firearm access.

## Key Points

**Question:**

What are the frequency, distribution, and factors associated with defensive gun use?

**Findings:**

In this survey study of 3000 adults with firearm access, most (91.7%) reported no lifetime history of defensive gun use, whereas many reported lifetime gun violence exposure. Individuals exposed to gun violence and who carried firearms more frequently and stored firearms loaded and unlocked were more likely to report prior defensive gun use.

**Meaning:**

These findings suggest that policy conversations that center the defensive gun use misstate the risk profile of firearm access.

## Teaser

This survey study examines the frequency and characteristics, including exposure to gun violence, associated with defensive gun use among US adults.

## Introduction

Defensive gun use (DGU) is a focus of firearm policy discussions. Most firearm owners report that self-defense is their primary reason for carrying firearms.^1^ Despite the importance of DGU in policy debates, there is a lack of consensus regarding its prevalence, with some studies suggesting significant overestimation in self-reported data.^2^ This study addresses this gap by examining the frequency, distribution, and correlates of self-reported DGU in a national sample of US adults with firearm access.

For decades, researchers have estimated the frequency of DGU. Some studies estimate between 100 000 and 500 000 events per year,^3,4^ while others have generated implausible overestimates of millions of instances per year^5,6^; however, the latter estimates have been criticized for methodological problems^2,7,8^ and undisclosed conflicts of interest. A 2024 study using 35 years of data from the National Crime Victimization Survey reported that individuals who experienced a crime reported DGU a consistent mean of 61 000 to 65 000 times per year across the study period.^9^ However, the study by McDowall et al^9^ only included officially reported incidents during a crime. Studies have found that criminal gun use is more common than DGU,^10^ with little evidence DGU prevents injury or property loss.^11^

In addition to the lack of clarity regarding the frequency of DGU, little is known about which individuals engage in DGU. It may be, for instance, that DGU is driven primarily by opportunities created through interactions with firearms. Individuals who have been injured or threatened with a firearm are more likely to own and carry firearms,^12^ potentially increasing opportunities or motivation for DGU. Additionally, carrying firearms and storing them unsecured are associated with increased risk of firearm injury and death^13^ and may correlate with higher rates of DGU due to increased accessibility.

Understanding demographic patterns in firearm behaviors is crucial for developing targeted interventions to reduce firearm mortality. Previous research has shown that men are more likely to carry firearms than women.^14^ Additionally, firearm ownership and carrying behaviors vary by race, ethnicity, and region of residence.^15,16^ However, little is known about how these demographic factors interact with DGU.

The objective of this study is to understand the prevalence of DGU and the experiences and characteristics associated with it. First, our study assesses the prevalence of 4 DGU responses to perceived threats (ie, telling them you have a firearm, showing them a firearm, firing near them, or firing at them) and the extent to which subgroups of firearm owners are more likely to have engaged in DGU. Second, we contrast the frequency of DGU with that of gun violence exposure (GVE). Given the well-documented consequences of GVE,^17,18^ this analysis provides a parallel model through which to contextualize DGU findings. Third, we examine demographic, community, and experiential factors associated with each form of DGU.

## Method

This survey study was approved by the Rutgers Biomedical and Health Sciences institutional review board and all participants provided informed consent. We followed the American Association for Public Opinion Research (AAPOR) reporting guideline in the design of our sample and survey instrument and for analysis and reporting of results.

### Participants and Procedures

A national sample was recruited May 15 to May 28, 2024, via Ipsos KnowledgePanel, a probability-based panel designed to be representative of US adults. The survey was sent via email to 12 822 adult panel members, 8647 of whom (67%) engaged with the informed consent and 8009 of whom (93%) consented to participate, completed the online survey, and were part of the final sample.

In all analyses, we used the subsample of 3018 participants (37.7%) who endorsed firearm access. An additional 18 individuals did not respond to items assessing DGU, resulting in a final sample size of 3000 adults.

Data weights were computed to reflect selection probabilities. Design weights were raked to geodemographic distributions based on the March 2023 supplement of the Current Population Survey and Pew’s 2023 National Public Opinion Reference Survey.

### Measures

#### Demographics

We leveraged demographic items from KnowledgePanel profiles, including gender, age, racial and ethnic identity, metropolitan area, education, region of residence, and household income. Race and ethnicity were collected by KnowledgePanel to describe the sample and categorized as Black, non-Hispanic; Hispanic; White, non-Hispanic; other, non-Hispanic; and 2 or more races.

#### DGU

Participants were asked “Have you ever engaged in any of the following behaviors with a firearm to protect yourself or someone else?” Answers included “Told someone who was threatening you that you had a firearm,” “Shown your firearm to someone who was threatening you,” “Fired your firearm in the vicinity of but not at someone who was threatening you,” “Fired your firearm at someone who was threatening you,” and “None of the above.” If an individual endorsed any of the first 4 responses, they were asked to indicate when the experience last occurred, facilitating the coding of lifetime and past-year experiences.

#### GVE

Participants were asked “Please indicate whether you have had each of the following experiences.” Answers included “Have you ever known someone personally that has died by suicide with a firearm?” “Have you ever been threatened with a firearm by another person?” “Have you ever been intentionally shot with a firearm by another person?” “Have you personally known someone, such as a friend or family member, who has been shot on purpose by another person with a firearm?” “Have you ever personally witnessed a shooting in the neighborhood where you lived at the time?” and “Have you ever heard gun shots in the neighborhood where you lived at the time?” If an individual endorsed any of responses, they were asked to indicate when the experience last occurred, facilitating the coding of lifetime and past-year exposure.

#### Risky Firearm Behaviors

Participants were asked “Are any of the firearms in your home typically stored both unlocked and loaded?” Furthermore, participants were asked “How frequently do you carry a firearm on your person outside of your home?” Answers were scored on a 6-point Likert scale ranging from 0, indicating never, to 5, always.

### Statistical Analysis

We ran a series of descriptive analyses to examine the frequency of lifetime and past-year DGU and GVE for both the full sample and various subgroups. To analyze variables associated with the likelihood of endorsing lifetime engagement in any of the specific DGU behaviors, we ran a series of 4 binary logistic regressions. Demographic variables, GVE, firearm carrying, and firearm storage served as independent variables in each equation, with each of the 4 DGU behaviors serving as the dependent variable per model and odds ratios (ORs) indicating the odds of positively endorsing DGU. *P* values were 2-sided, and statistical significance was set at *P* < .05. Analyses were conducted using SPSS version 29.0.1.0 (IBM) from July to September 2024.

## Results

A total of 3000 adults (532 [51.1%] male; 982 [32.7%] aged ≥60 years) endorsed firearm access and responded to DGU items, including 295 Black, non-Hispanic participants (9.8%); 365 Hispanic participants (12.2%); and 2178 White, non-Hispanic participants (72.6%). Sample demographics can be found in Table 1.

**Table 1.** Sample Demographics

Characteristic	Participants, No. (%)
Gender	
Male	1532 (51.1)
Female	1467 (48.9)
Race and ethnicity	
Black, non-Hispanic	295 (9.8)
Hispanic	365 (12.2)
White, non-Hispanic	2178 (72.6)
Other, non-Hispanic	113 (3.8)
≥2 Races, non-Hispanic	49 (1.6)
Age, y	
18-29	509 (17.0)
30-44	725 (24.2)
45-59	785 (26.1)
≥60	982 (32.7)
Education	
No high school diploma or GED	224 (7.5)
High school graduate	896 (29.9)
Some college or Associate’s degree	905 (30.2)
≥Bachelor’s degree	975 (32.5)
Household income, $	
<10 000	49 (1.6)
10 000-24 999	138 (4.6)
25 000-49 999	377 (12.6)
50 000-74 999	487 (16.2)
75 000-99 999	434 (14.5)
100 000-149 999	680 (22.7)
≥150 000	837 (27.9)
Metro status	
Nonmetro	610 (20.3)
Metro	2390 (79.7)
Region of residence	
Northeast	352 (11.7)
Midwest	663 (22.1)
South	1333 (44.4)
West	652 (21.7)
Political beliefs	
Highly conservative	477 (15.9)
Somewhat conservative	864 (28.8)
Moderate	1155 (38.4)
Somewhat liberal	334 (11.1)
Highly liberal	147 (4.9)
Refused to answer	22 (0.7)

Abbreviation: GED, general educational development.

### Frequency of DGU

Most participants with firearm access endorsed never having engaged in DGU (2750 participants [91.7%; 95% CI, 90.6%-92.7%]). This percentage was relatively similar across subgroups, with few exceptions. Among 556 respondents who had been threatened with a firearm, 73.7% (95% CI, 69.8%-77.3%) endorsed never having engaged in DGU, and among 64 respondents who had previously been shot, 43.6% (95% CI, 31.4%-56.5%) endorsed never having engaged in DGU.

Among 250 participants (8.3%) who reported DGU, the most frequently endorsed form of DGU was showing a firearm to a perceived threat (142 participants [4.7%]). The second most common was telling a perceived threat they have a firearm (113 participants [3.8%]). Only 1.1% (95% CI, 0.8%-1.6%) of the sample endorsed having fired in the vicinity of but not at a threat and only 1.2% (95% CI, 0.9%-1.7%) endorsed having fired at a threat. These percentages were also similar across subgroups, with the exception of those with GVE. For instance, of 22 participants who had previously been shot, 34.2% (95% CI, 23.1%-47.2%) endorsed having fired at a threat. Therefore, 59.5% of the instances of shooting a firearm at a threat occurred among individuals who had previously been shot despite such individuals accounting for only 2.1% of the sample.

Instances of past-year DGU were rare, with less than 1% of the sample endorsing any of the behaviors and only 3 individuals endorsing having shot their firearm at a threat within the past year. Full results for DGU frequency are presented in Table 2.

**Table 2.** Frequency of Defensive Gun Use Within the Full Sample and Subgroups of Firearm Owners

Characteristic	Sample size	Defensive gun use, No. (%) [95% CI]								
		Lifetime					Past year			
		Told perceived threat about firearm	Showed perceived threat firearm	Fired in vicinity of but not at perceived threat	Fired at perceived threat	None of the above	Told perceived threat about firearm	Showed perceived threat firearm	Fired in vicinity of but not at perceived threat	Fired at perceived threat
Full sample	3000	113 (3.8) [3.2-4.6]	142 (4.7) [4.0-5.5]	32 (1.1) [0.8-1.6]	37 (1.2) [0.9-1.7]	2750 (91.7) [90.6-92.7]	20 (0.7) [0.5-1.1]	20 (0.7) [0.5-1.1]	9 (0.3) [0.2-0.6]	5 (0.2) [0.1-0.5]
Been threatened with a firearm	556	66 (11.9) [9.4-14.9]	84 (15.0) [12.2-18.3]	16 (2.9) [1.7-4.8]	29 (6.4) [4.6-8.9]	410 (73.7) [69.8-77.3]	12 (2.2) [1.2-3.9]	12 (2.2) [1.2-3.9]	19 (3.4) [2.1-5.4]	3 (0.5) [0.1-1.7]
Been shot	64	10 (14.9) [7.6-26.5]	16 (24.7) [15.1-37.3]	10 (15.1) [7.8-26.7]	22 (34.2) [23.1-47.2]	28 (43.6) [31.4-56.5]	1 (1.6) [0.1-9.6]	5 (7.8) [2.9-18.0]	5 (7.8) [2.9-18.0]	3 (4.7) [1.2-14.0]
Store firearm loaded and unlocked	875	52 (5.9) [4.5-7.7]	78 (8.9) [7.1-11.0]	20 (2.3) [1.5-3.6]	22 (2.5) [1.6-3.8]	748 (85.5) [83.0-87.7]	12 (1.4) [0.8-2.5]	11 (1.3) [0.7-2.4]	6 (0.7) [0.2-1.6]	3 (0.3) [0.1-1.0]
Race and ethnicity										
Black	295	15 (5.1) [3.0-8.4]	13 (4.5) [2.5-7.7]	8 (2.8) [1.3-5.6]	7 (2.4) [1.1-5.1]	263 (89.0) [84.7-92.2]	5 (1.7) [0.6-4.1]	5 (1.7) [0.6-4.1]	4 (1.4) [0.5-3.7]	1 (0.3) [<0.1-2.1]
White	2178	71 (3.3) [2.6-4.2]	96 (4.4) [3.6-5.4]	15 (0.7) [0.4-1.2]	20 (0.9) [0.6-1.4]	2021 (92.8) [91.6-93.8]	11 (0.5) [0.3-0.9]	11 (0.5) [0.3-0.9]	3 (0.1) [<0.1-0.4]	0
Metro status										
Metro	2390	89 (3.7) [3.0-4.6]	113 (4.7) [3.9-5.7]	26 (1.1) [0.7-1.6]	29 (1.2) [0.8-1.7]	2190 (91.6) [90.4-92.7]	15 (0.6) [0.3-1.0]	12 (0.5) [0.3-0.9]	8 (0.3) [0.1-0.6]	3 (0.1) [<0.1-0.4]
Nonmetro	610	24 (2.2) [1.2-3.8]	29 (4.8) [3.3-6.9]	6 (0.9) [0.4-2.1]	7 (1.2) [0.5-2.5]	560 (91.7) [89.2-93.7]	4 (0.7) [0.2-1.9]	7 (1.1) [0.5-2.4]	1 (0.2) [<0.1-1.1]	1 (0.2) [<0.1-1.1]
Age, y										
18-29	509	24 (4.6) [3.0-6.9]	23 (4.6) [3.0-6.9]	1 (0.2) [<0.1-1.3]	6 (1.1) [0.4-2.6]	462 (90.7) [87.8-93.0]	7 (0.5) [0.1-1.7]	7 (0.5) [0.1-1.7]	0	3 (0.2) [<0.1-1.3]
30-44	726	28 (3.9) [2.7-5.6]	32 (4.4) [3.1-6.2]	11 (1.5) [0.8-2.8]	7 (1.0) [0.4-2.1]	665 (91.7) [89.3-93.5]	7 (1.0) [0.4-2.1]	7 (1.0) [0.4-2.1]	8 (1.0) [0.5-2.3]	1 (0.2) [<0.1-0.9]
45-59	788	34 (4.4) [3.1-6.0]	44 (5.6) [4.1-7.5]	8 (1.0) [0.5-2.1]	9 (1.2) [0.6-2.2]	712 (90.4) [88.0-92.3]	4 (0.4) [0.1-1.4]	4 (0.4) [0.1-1.4]	1 (0.1) [<0.1-0.8]	0
≥60	989	27 (2.7) [1.8-4.0]	43 (4.3) [3.2-5.9]	12 (1.2) [0.7-2.2]	15 (1.5) [0.9-2.6]	911 (92.1) [90.2-93.7]	3 (0.3) [0.1-1.0]	2 (0.2) [<0.1-0.8]	1 (0.1) [<0.1-0.7]	0
Census region										
Northeast	352	13 (3.6) [2.0-6.3]	16 (4.4) [2.6-7.3]	6 (1.6) [0.6-3.7]	2 (0.6) [0.1-2.3]	325 (92.3) [88.9-94.8]	0	2 (0.1) [<0.1-1.5]	3 (0.2) [<0.1-1.7]	0
Midwest	663	18 (2.7) [1.7-4.3]	20 (3.0) [1.9-4.7]	4 (0.6) [0.2-1.6]	4 (0.6) [0.2-1.6]	625 (94.2) [92.1-95.8]	4 (0.2) [<0.1-1.1]	2 (0.1) [<0.1-0.9]	0	0
South	1333	54 (4.0) [3.0-5.2]	72 (5.4) [4.3-6.8]	16 (1.2) [0.7-2.0]	23 (1.7) [1.1-2.6]	1208 (90.6) [88.9-92.1]	11 (0.3) [<0.1-0.8]	14 (0.4) [0.2-1.0]	4 (0.1) [<0.1-0.5]	5 (0.1) [<0.1-0.5]
West	652	29 (4.4) [3.0-6.3]	35 (5.3) [3.8-7.4]	6 (0.9) [0.4-2.1]	7 (1.1) [0.5-2.3]	592 (90.9) [88.4-92.9]	5 (0.3) [0.1-1.2]	2 (<0.1) [<0.1-0.9]	2 (<0.1) [<0.1-0.9]	0
Gender										
Male	1534	81 (5.3) [4.3-6.6]	105 (6.9) [5.7-8.3]	20 (1.3) [0.8-2.0]	29 (1.9) [1.3-2.8]	1361 (88.7) [87.0-90.2]	17 (0.5) [0.2-1.0]	15 (0.4) [0.2-0.9]	6 (0.1) [<0.1-0.5]	3 (0.1) [<0.1-0.5]
Female	1467	33 (2.2) [1.5-3.1]	35 (2.4) [1.7-3.4]	12 (0.8) [0.4-1.5]	8 (0.5) [0.2-1.1]	1390 (94.7) [93.4-95.8]	3 (<0.1) [<0.1-0.4]	5 (0.1) [<0.1-0.5]	4 (0.1) [<0.1-0.5]	1 (<0.1) [<0.1-0.4]

### Frequency of GVE

To contextualize the DGU findings, we examined the percentage of the full sample of adults with firearm access and subsamples that endorsed GVE. In total, 1033 individuals (34.4%) with firearm access endorsed ever personally knowing someone who had died by firearm suicide, and 74 individuals (3.2%) endorsed having known someone who died by firearm suicide in the past year. There was substantial variability in these percentages across subgroups. For instance, among individuals who reported having been shot, 62.8% (95% CI, 49.8%-74.3%) endorsed knowing someone who had died by firearm suicide, with 15.6% (95% CI, 8.1%-27.3%) reporting this happened within the past year.

Most participants (51.8%; 95% CI, 50.0%-53.6%) with firearm access reported having heard gunshots in their neighborhood, 21.3% (95% CI, 19.9%-22.8%) of participants reported knowing someone who had been shot, 18.5% (95% CI, 17.2%-20.0%) reported having been threatened with a firearm, 8.6% (95% CI, 7.6%-9.7%) reported having witnessed a shooting in their neighborhood, and 2.1% (95% CI, 1.6%-2.8%) reported having been shot. Within the past year, 32.7% (95% CI, 31.0%-34.4%) reported having heard gunshots in their neighborhood, 1.9% (95% CI, 1.5%-2.5%) reported knowing someone who had been shot, 1.3% (95% CI, 0.9%-1.8%) endorsed having been threatened with a firearm, 1.2% (95% CI, 0.8%-1.7%) reported having witnessed a shooting in their neighborhood, and 0.2% (95% CI, <0.1%-0.5%) reported having been shot. Here again, there was variability across subgroups. Of note, Black, non-Hispanic firearm owners reported higher rates across almost all variables. For instance, in terms of lifetime exposure, 68.8% (95% CI, 63.1%-74.0%) Black, non-Hispanic participants reported having heard gunshots in their neighborhood (46.4% [95% CI, 40.6%-52.3%] in the past year), 47.6% (95% CI, 41.8%-53.5%) reported knowing someone who had been shot (9.5% [95% CI, 6.5%-13.6%] in the past year), 27.7% (95% CI, 22.8%-33.3%) reported having been threatened with a firearm (4.1% [95% CI, 0.2-7.2] in the past year), and 17.8% (95% CI, 13.7%-22.8%) reported having witnessed a shooting in their neighborhood (3.7% [95% CI, 2.0%-6.7%] in the past year). Full results for GVE can be found in Table 3. DGU frequency relative to GVE is displayed in the Figure.

**Table 3.** Frequency of Gun Exposures Within the Full Sample and Subgroups of Firearm Owners

Characteristic	Sample size	Gun violence exposure, No. (%) [95% CI]											
		Lifetime						Past year					
		Known someone who died by firearm suicide	Threatened with a firearm	Shot by a firearm	Known someone shot by a firearm	Witnessed a shooting in neighborhood	Heard gunshots in neighborhood	Known someone who died by firearm suicide	Threatened with a firearm	Shot by a firearm	Known someone shot by a firearm	Witnessed a shooting in neighborhood	Heard gunshots in neighborhood
Full sample	3000	1033 (34.4) [32.7-36.1]	556 (18.5) [17.2-20.0]	64 (2.1) [1.6-2.8]	640 (21.3) [19.9-22.8]	257 (8.6) [7.6-9.7]	1553 (51.8) [50.0-53.6]	74 (3.2) [2.6-3.9]	40 (1.3) [0.9-1.8]	7 (0.2) [<0.1-0.5]	57 (1.9) [1.5-2.5]	37 (1.2) [0.8-1.7]	981 (32.7) [31.0-34.4]
Been threatened with a firearm	556	270 (48.5) [44.3-52.7]	556 (100.0) [NA]	48 (8.6) [6.5-11.3]	290 (52.1) [47.9-56.3]	145 (26.1) [22.5-30.0]	416 (75.0) [71.1-78.5]	23 (4.1) [2.7-6.2]	40 (7.2) [5.3-9.8]	4 (0.7) [0.2-1.9]	34 (6.1) [4.3-8.5]	20 (3.6) [2.3-5.6]	259 (46.6) [42.4-50.9]
Been shot	64	40 (62.8) [49.8-74.3]	48 (74.7) [62.2-84.5]	64 (100.0) [NA]	46 (72.2) [59.4-82.3]	26 (40.5) [28.6-53.5]	41 (64.9) [51.9-76.1]	10 (15.6) [8.1-27.3]	6 (9.4) [3.9-20.0]	7 (10.9) [4.9-21.8]	8 (12.5) [5.9-23.7]	4 (6.3) [2.0-16.1]	22 (34.4) [23.3-47.4]
Firearm stored loaded and unlocked	875	352 (40.2) [36.9-43.5]	232 (26.5) [23.6-30.0]	36 (4.1) [2.9-5.7]	239 (27.4) [24.5-30.5]	98 (11.2) [9.2-13.5]	512 (58.5) [55.2-61.8]	33 (3.8) [2.7-5.4]	22 (2.5) [1.6-3.8]	7 (0.8) [0.3-1.7]	26 (3.0) [2.0-4.4]	17 (1.9) [1.1-3.1]	337 (38.5) [35.3-41.8]
Race and ethnicity													
Black	295	73 (24.7) [20.0-30.1]	82 (27.7) [22.8-33.3]	11 (3.8) [2.0-6.9]	141 (47.6) [41.8-53.5]	53 (17.8) [13.7-22.8]	203 (68.8) [63.1-74.0]	6 (2.0) [0.8-4.6]	12 (4.1) [0.2-7.2]	2 (0.7) [0.1-2.7]	28 (9.5) [6.5-13.6]	11 (3.7) [2.0-6.7]	137 (46.4) [40.6-52.3]
White	2178	807 (37.1) [35.1-39.2]	348 (16.0) [14.5-17.6]	31 (1.4) [1.0-2.0]	365 (16.7) [15.2-18.4]	117 (5.4) [4.5-6.5]	1063 (48.8) [46.7-50.9]	49 (2.2) [1.6-2.9]	14 (0.6) [0.3-1.1]	1 (<0.1) [<0.1-0.3]	15 (0.7) [0.4-1.2]	10 (0.5) [0.2-0.9]	676 (31.0) [29.1-33.0]
Metro status													
Metro	2390	784 (32.8) [30.9-34.7]	440 (18.4) [16.9-20.0]	48 (2.0) [1.5-2.7]	511 (21.4) [19.8-23.1]	220 (9.2) [8.1-10.5]	1235 (51.7) [49.7-53.7]	56 (2.3) [1.8-3.0]	31 (1.3) [0.9-1.9]	6 (0.3) [0.1-0.6]	48 (2.0) [1.5-2.7]	35 (1.5) [1.1-2.1]	759 (31.8) [29.9-33.7]
Nonmetro	610	249 (40.8) [36.9-44.8]	116 (19.1) [16.1-22.5]	16 (2.6) [1.5-4.3]	129 (21.1) [18.0-24.6]	37 (6.1) [4.4-8.3]	318 (52.1) [48.1-56.1]	16 (2.6) [1.5-4.3]	9 (1.5) [0.7-2.9]	1 (0.2) [<0.1-1.1]	9 (1.5) [0.7-2.9]	2 (0.3) [0.0-1.3]	225 (36.9) [33.1-40.9]
Age, y													
18-29	509	98 (19.2) [15.9-23.0]	83 (16.3) [13.3-19.9]	4 (0.7) [0.2-2.0]	97 (19.1) [15.8-22.9]	59 (11.6) [9.0-14.8]	275 (54.2) [49.8-59.6]	7 (1.4) [0.6-3.0]	12 (2.4) [1.3-4.3]	0	20 (3.9) [2.5-6.1]	13 (2.6) [1.5-5.0]	173 (34.0) [29.9-38.3]
30-44	726	232 (32.0) [28.6-35.5]	146 (20.1) [17.3-23.3]	19 (2.6) [1.6-4.1]	188 (25.9) [22.8-29.3]	70 (9.7) [7.6-12.0]	388 (53.5) [49.7-57.1]	25 (3.4) [2.3-5.1]	21 (2.8) [1.9-4.5]	3 (0.5) [0.1-1.3]	14 (1.9) [1.1-3.3]	11 (1.6) [0.8-2.8]	243 (33.5) [30.1-37.1]
45-59	788	300 (38.1) [34.7-41.6]	178 (22.6) [19.8-25.7]	20 (2.5) [1.6-4.0]	170 (21.6) [18.8-24.7]	82 (10.4) [8.4-12.8]	437 (55.4) [51.9-59.0]	23 (3.0) [1.9-4.4]	9 (1.0) [0.6-2.2]	3 (0.4) [0.1-1.2]	16 (2.1) [1.2-3.4]	11 (1.3) [0.7-2.6]	269 (34.2) [30.9-37.6]
≥60	989	410 (41.4) [38.4-44.6]	154 (15.6) [13.4-18.0]	21 (2.2) [1.4-3.3]	189 (19.1) [16.7-21.7]	48 (4.9) [3.6-6.4]	460 (46.5) [43.4-49.7]	18 (1.9) [1.1-2.9]	2 (0.2) [<0.1-0.8]	1 (0.1) [<0.1-0.7]	8 (0.8) [0.4-1.7]	3 (0.3) [0.1-1.0]	301 (30.5) [27.6-33.4]
Census region													
Northeast	352	106 (30.0) [25.3-35.1]	49 (13.9) [10.6-18.1]	10 (2.9) [1.5-5.4]	49 (13.9) [10.6-18.1]	23 (6.6) [4.3-9.9]	143 (40.6) [35.5-46.0]	9 (2.6) [1.3-5.0]	2 (0.6) [0.1-2.3]	3 (0.9) [0.2-2.8]	4 (1.1) [0.4-3.0]	4 (1.1) [0.4-3.0]	98 (27.8) [23.3-32.9]
Midwest	663	252 (38.0) [34.3-41.8]	111 (16.8) [14.1-19.9]	11 (1.6) [0.8-3.0]	122 (18.4) [15.6-21.6]	61 (9.2) [7.2-11.7]	315 (47.5) [43.7-51.4]	20 (3.0) [1.9-4.7]	7 (1.1) [0.5-2.3]	0	12 (1.8) [1.0-3.2]	7 (1.1) [0.5-2.3]	199 (30.0) [26.6-33.7]
South	1333	453 (34.0) [31.4-36.6]	283 (21.3) [19.1-23.6]	33 (2.5) [1.8-3.5]	326 (24.5) [22.2-26.9]	105 (7.9) [6.5-9.5]	756 (56.7) [54.9-60.3]	33 (2.5) [1.8-3.5]	22 (1.7) [1.1-2.6]	4 (0.3) [0.1-0.8]	34 (2.6) [1.8-3.6]	17 (1.3) [0.8-2.1]	501 (37.6) [35.0-40.3]
West	652	222 (34.1) [30.5-37.9]	113 (17.3) [14.5-20.5]	10 (1.5) [0.8-2.9]	143 (21.9) [18.8-25.3]	67 (10.3) [8.1-13.0]	339 (52.0) [48.1-55.9]	13 (2.0) [1.1-3.5]	9 (1.4) [0.7-2.7]	0	5 (0.8) [0.3-1.9]	9 (1.4) [0.7-2.7]	184 (28.2) [24.8-31.9]
Defensive gun use	113												
Told threat about firearm	113	56 (49.1) [39.6-58.6]	66 (58.6) [48.9-67.7]	10 (8.4) [4.2-15.5]	66 (58.8) [49.1-67.9]	35 (31.1) [22.9-40.6]	83 (73.4) [64.1-81.1]	1 (0.9) [0.1-5.6]	8 (7.1) [3.3-13.9]	1 (0.9) [0.1-5.6]	6 (5.3) [2.2-11.7]	5 (4.4) [1.6-10.5]	54 (47.8) [38.4-57.4]
Showed firearm to threat	113	66 (46.3) [38.0-54.8]	84 (59.0) [50.4-67.1]	16 (11.1) [6.7-17.7]	78 (55.0) [456.4-63.3]	32 (22.9) [16.5-30.9]	96 (67.9) [59.5-75.3]	2 (1.4) [0.2-5.5]	8 (5.6) [2.6-11.1]	2 (1.4) [0.2-5.5]	9 (6.3) [3.1-12.0]	7 (4.9) [2.2-10.2]	65 (45.8) [37.5-54.3]
Shot in vicinity of threat	113	18 (55.1) [36.8-72.2]	16 (51.0) [33.1-68.6]	10 (30.3) [16.0-49.2]	17 (53.1) [35.0-70.5]	15 (46.6) [29.3-64.7]	19 (60.5) [41.9-76.7]	4 (12.5) [4.1-29.9]	4 (12.5) [4.1-29.9]	5 (15.6) [5.9-33.5]	5 (15.6) [5.9-33.5]	8 (25.0) [12.1-43.8]	11 (34.4) [19.2-53.3]
Shot at threat	113	26 (70.1) [52.7-83.4]	35 (96.7) [83.3-99.7]	22 (59.4) [42.1-74.8]	30 (82.0) [65.3-92.1]	14 (37.5) [22.7-54.9]	26 (72.1) [54.7-85.0]	5 (13.5) [5.1-29.6]	6 (16.2) [6.8-32.7]	3 (8.1) [2.1-23.0]	6 (16.2) [6.8-32.7]	2 (5.4) [0.9-19.5]	16 (43.2) [27.5-60.3]
Gender													
Male	1534	543 (35.4) [33.0-37.9]	383 (25.0) [22.9-27.3]	51 (3.4) [2.6-4.5]	393 (25.6) [23.5-27.9]	168 (11.0) [9.5-12.7]	826 (53.9) [51.4-56.4]	34 (2.2) [1.6-3.1]	25 (1.6) [1.1-2.4]	4 (0.3) [0.1-0.8]	30 (2.0) [1.4-2.9]	20 (1.3) [0.9-2.0]	501 (32.6) [30.3-35.0]
Female	1467	490 (33.4) [31.0-35.9]	173 (11.8) [10.2-13.6]	12 (0.8) [0.4-1.5]	247 (16.8) [14.9-18.8]	89 (6.1) [5.0-7.5]	727 (49.6) [47.0-52.2]	40 (2.7) [20-3.7]	15 (1.0) [0.5-1.7]	3 (0.2) [<0.1-0.6]	26 (1.8) [1.2-2.7]	17 (1.2) [0.7-1.9]	482 (32.9) [30.5-35.4]

Abbreviation: NA, not applicable.

**Figure.**
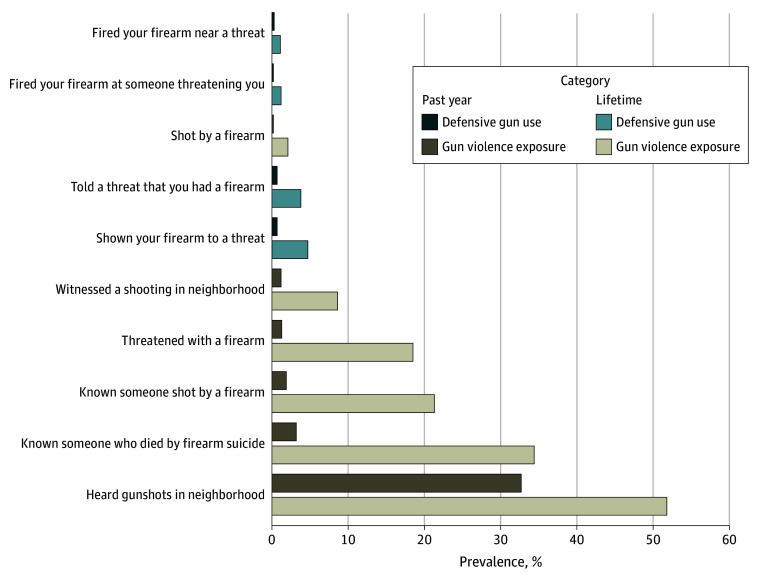
Prevalence and Frequency of Firearm-Related Incidents Among Adults With Firearm Access

### Factors Associated With DGU

In our final analysis, we examined which variables were associated with lifetime endorsement of each form of DGU. Demographics were largely not associated with DGU (Table 4). The only exception was that female respondents were less likely than male respondents to have shown their firearm to someone threatening them (OR, 0.74 [95% CI, 0.47-1.16]).

**Table 4.** Binary Logistic Regressions Examining Factors Associated With Lifetime Defensive Gun Use

Characteristic	Told threat you have firearm		Shown firearm to threat		Fired in vicinity of threat		Fired at threat	
	OR (95% CI)	*P* value	OR (95% CI)	*P* value	OR (95% CI)	*P* value	OR (95% CI)	*P* value
Female gender (vs male)	0.74 (0.47-1.16)	.19	0.63 (0.41-0.98)	.04	1.03 (0.46-2.31)	.95	1.33 (0.48-3.69)	.58
Race (ref: White)		.95		.28		.48		.74
Black	0.79 (0.42-1.48)	.46	0.61 (0.32-1.17)	.14	2.35 (0.88-6.26)	.09	1.58 (0.54-4.68)	.41
White	1 [Reference]	NA	1 [Reference]	NA	1 [Reference]	NA	1 [Reference]	NA
Other	1.00 (0.33-3.01)	.99	1.72 (0.70-4.21)	.24	1.04 (0.09-11.88)	.97	NA	.99
Hispanic	0.93 (0.51-1.69)	.82	0.91 (0.52-1.60)	.74	1.16 (0.39-3.47)	.79	0.67 (0.20-2.22)	.52
≥2 Races	1.18 (0.33-4.18)	.80	1.67 (0.55-5.08)	.37	2.47 (0.34-17.96)	.37	2.09 (0.27-16.39)	.49
Metropolitan area (vs nonmetropolitan)	0.94 (0.57-1.55)	.80	1.06 (0.66-1.71)	.80	1.05 (0.40-2.75)	.93	0.92 (0.35-2.43)	.87
Education	1.09 (0.86-1.39)	.47	1.16 (0.93-1.46)	.19	1.06 (0.69-1.64)	.78	1.26 (0.75-2.13)	.38
Household income^a^	1.00 (0.88-1.14)	.97	1.09 (0.96-1.24)	.18	0.92 (0.73-1.17)	.51	1.07 (0.82-1.40)	.61
Threatened by firearm	2.73 (1.70-4.36)	<.001	2.99 (1.95-4.57)	<.001	1.56 (0.62-3.93)	.34	47.23 (7.09-314.71)	<.001
Shot by firearm	1.16 (0.53-2.54)	.72	1.97 (0.99-3.92)	.05	7.52 (2.89-19.61)	<.001	30.24 (12.33-74.18)	<.001
Known someone shot	2.33 (1.45-3.75)	<.001	2.08 (1.34-3.21)	<.001	1.16 (0.44-3.09)	.76	2.47 (0.88-6.97)	.09
Heard gunshots	1.24 (0.76-2.01)	.40	1.07 (0.69-1.64)	.77	0.42 (0.16-1.09)	.07	0.75 (0.27-2.08)	.58
Witnessed shooting	2.01 (1.18-3.41)	.01	1.24 (0.73-2.13)	.43	5.66 (2.06-15.54)	<.001	1.11 (0.41-2.98)	.84
Firearm carrying frequency	1.30 (1.16-1.47)	<.001	1.49 (1.34-1.67)	<.001	1.10 (0.88-1.38)	.40	1.51 (1.18-1.93)	.001
Firearm stored loaded and unlocked firearm	1.10 (0.72-1.70)	.66	1.58 (1.06-2.34)	.03	2.70 (1.21-6.01)	.02	1.33 (0.57-3.11)	.52

Abbreviations: NA, not applicable; OR, odds ratio.

^a^ Household income was grouped as less than $10 000, $10 000 to $24 999, $25 000 to $49 999, $50 000 to $74 999, $75 000 to $99 999, $100 000 to $149 999, and $150 000 or more.

The most consistent and robust associations with DGU involved GVE and firearm carrying and storage. Specifically, individuals who had been threatened with a firearm were more likely to have told a perceived threat about their firearm (OR, 2.73 [95% CI, 1.70-4.36]), to have shown their firearm to a perceived threat (OR, 2.99 [95% CI, 1.95-4.57]), and to have shot at a perceived threat (OR, 47.23 [95% CI, 7.09-314.71]). Individuals who had been shot were more likely to have fired in the vicinity of but not at a perceived threat (OR, 7.52 [95% CI, 2.89-19.61]) and to have shot at a perceived threat (OR, 30.24 [95% CI, 12.33-74.18]). Individuals who knew someone who had been shot were more likely to have told a perceived threat about their firearm (OR, 2.33 [95% CI, 1.45-3.75]) and to have shown their firearm to a perceived threat (OR, 2.08 [95% CI, 1.34-3.21]). Individuals who had heard gunshots in their neighborhood were more likely to have told a perceived threat about their firearm (OR, 2.01 [95% CI, 1.18-3.41]) and to have fired in the vicinity of but not at a perceived threat (OR, 5.66 [95% CI, 2.06-15.54]).

Participants who reported greater frequency of firearm carrying were more likely to have told a perceived threat about their firearm (OR, 1.30 [95% CI, 1.16-1.47]), to have shown their firearm to a perceived threat (OR, 1.49 [95% CI, 1.34-1.67]), and to have shot at a perceived threat (OR, 1.51 [95% CI, 1.18-1.93]). Lastly, participants who typically stored at least 1 firearm loaded and unlocked were more likely to have shown their firearm to a perceived threat (OR, 1.58 [95% CI, 1.06-2.34]) and to have shot in the vicinity of but not at a perceived threat (OR, 2.70 [95% CI, 1.21-6.01]).

## Discussion

Our survey study leveraged nationally representative data collected in 2024 to estimate the frequency of 4 types of DGU. Additionally, we contrasted our findings with the frequency of GVE to provide a context for the occurrence of DGU and analyzed factors associated with lifetime DGU. Our results produced 3 main findings.

First, DGU is rare—approximately 92% of participants with firearm access said they had never used a gun defensively. Approximately 0.7% of participants had told someone that they had a firearm or shown their firearm to a perceived threat within the past year. Approximately 0.3% of participants had fired in the vicinity of a threat, whereas about 0.2% of participants had fired at a perceived threat in the past year. There are approximately 260 million adults in the US, and our results suggest approximately 38% have access to a firearm in the home, cohering with other estimates.^19^ Taken together, this means approximately 97.8 million US adults have household firearm access, which equates to approximately 195 600 instances of DGU per year in which someone fired at a perceived threat. When including the additional 0.3% of respondents who said they fired in the vicinity of but not at a threat, the annual estimate of DGU in which a gun is fired totals approximately 489 000 events per year. This estimate is higher than recent studies using National Crime Victimization Survey data (61 000-65 000 events per year) and the Gun Violence Archive (386 justifiable firearm homicides per year),^6,20^ yet lower than recent survey estimates (several million).^10,21^

Second, consistent with prior reports,^22^ DGU is rare relative to GVE. For instance, approximately 33% of the sample indicated they had heard gunshots in their neighborhood within the past year, equating to approximately 32 million people. Approximately 3% of the sample, representing 2.9 million people, knew someone who died by firearm suicide in the past year, while the equivalent of 2 million people knew someone who had been shot by another person. Approximately 0.2% of the sample said they had been shot in the past year, equal to the proportion of respondents saying they had fired at a perceived threat. This elevated number suggests several possibilities, including that many nonfatal injuries go unreported to health care agencies, that data quality in health care systems is poor, that individuals with mild injuries do not present to health care settings, or that our data include false positives.

In general, DGU was elevated among people with GVE. It is worth re-emphasizing that approximately 60% of all instances of firing at a perceived threat occurred among the approximately 2% of the sample who had previously been shot, underscoring a significant overlap between shooting at a threat and having been shot and mirroring what has been documented in criminology literature.^23^ Overall, the results emphasize the significant vicarious toll of gun violence in the US that far outweighs the frequency of DGU.^24^ Our sample yielded sufficient incidents of some outcomes (eg, firearm suicide loss) to facilitate reliable population estimates. For others—particularly DGU and having been shot—the outcomes were rare enough to severely limit our confidence in the reliability of the estimates. This underscores our second point—that DGU was rare relative GVE, highlighting that narratives centering DGU as a consideration in firearm regulation and access misrepresent firearm risk profiles.

Overall, the most consistent factors associated with DGU included GVE and easier availability of firearms through carrying and storage. Research demonstrates that people are more sensitive to threats when they have experienced violence and that male firearm owners exhibit greater reactivity to unpredictable threats.^25,26^ Perceived threats may not always necessitate firearm use, but risk might appear more salient when people are primed by previous GVE and easier firearm access. This can potentially generate unnecessary instances of DGU that cause harm beyond the intention to stop a threat (eg, a bystander is shot) or result from an individual perceiving a nonthreatening person as an imminent threat. Given that personal protection is the primary reason for firearm ownership, people may be more likely to own and use firearms in a context of preexisting threat perception^27^ that is then further amplified by the existence of actual threats (eg, GVE) and easier availability of firearms through frequent carrying and unsecure storage.

It is also worth noting that the degree to which DGU incidents are necessary or beneficial remains unclear. Perceptions of threat do not equate to the actual presence of a threat necessitating self-defense. Furthermore, when self-defense is needed, a firearm may not be required. In the absence of a firearm, individuals may defend themselves in other ways—a point highlighted by the fact that other nations with fewer firearms are not disproportionately inundated with individuals injured due to their lack of firearm access. Indeed, given the overlap between GVE with DGU, it may be that DGU enhances the odds of firearm mortality rather than preventing it.

### Limitations

There are limitations to note for this study. First, despite the nationally representative nature of our data, we are cautious in our extrapolations, since estimates of DGU are based on survey item responses in a single sample. Second, our estimates may be subject to self-reporting bias, such that individuals inflate the frequency of DGU or misperceive the need for DGU. Furthermore, although our full sample mirrors the demographic composition of US adults, the subsample in these analyses instead reflects that of individuals with home firearm access. Third, the wording of our items could have potentially resulted in modestly reduced rates of endorsement. For instance, if DGU occurred because an individual was threatening a respondent’s friend, they may have said “no,” because the item referred to individuals threatening “you.” Additionally, because our sample included only those with firearm access, the demographic composition skewed toward White individuals in the South and was older and more conservative than the general population. Furthermore, our analyses are cross-sectional, so we cannot determine causal relationships.

## Conclusions

The findings of this survey study provide a nuanced and representative understanding of how frequently various forms of DGU occur and which individuals are most likely to engage in DGU. Additionally, by providing this information alongside the frequency of GVE, our findings contextualize the occurrence of DGU, highlighting the extent to which firearms serve ostensibly protective and harmful functions. Reducing gun violence and the perceived risk for victimization can have the benefit of limiting DGU that may have unintended consequences in both private and public spheres by reducing perceptions of threat. Enhancements to firearm safety, including promoting secure storage and limiting carrying, may similarly reduce DGU. Of primary importance will be efforts to shift the narrative around firearms to deemphasize DGU as a common outcome. In doing so, policy efforts can be decoupled from efforts to prioritize safety through a lens of self-defense and instead center on efforts to reduce the risk of injury and death associated with firearm access.

## Notes

Supplement.
Data Sharing Statement
