# Supplementary material for: Lifetime and Past-Year Defensive Gun Use
Source: JAMA Netw Open. 2025 Mar 14;8(3):e250807. doi: 10.1001/jamanetworkopen.2025.0807 (PMC11909608; doi:10.1001/jamanetworkopen.2025.0807)
Supplement: Supplement. — Data Sharing Statement [file jamanetwopen-e250807-s001.pdf]

Data Sharing Statement

Data
Data available: Yes
Data types: Deidentified participant data
How to access data: mda141@sph.rutgers.edu
When available: With publication

Supporting Documents
Document types: Informed consent form
How to access documents: mda141@sph.rutgers.edu
When available: With publication

Additional Information
Who can access the data: Researchers whose proposed use of the data has been approved
Types of analyses: For a specified purpose
Mechanisms of data availability: After approval of a proposal
